# Cefoxitin inhibits the formation of biofilm involved in antimicrobial resistance MDR *Escherichia coli*

**DOI:** 10.1080/10495398.2025.2480176

**Published:** 2025-03-23

**Authors:** Hailan Ma, Dacheng Liu, Chen Song, Hongliang Fan, Weiguang Zhou, Hongxia Zhao

**Affiliations:** a College of Veterinary Medicine, Inner Mongolia Agricultural University, Huhhot, People’s Republic of China; b National Center of Technology Innovation for Dairy, Huhhot, People’s Republic of China; c Inner Mongolia Yili Industrial Group Co. Ltd, Huhhot, People’s Republic of China

**Keywords:** Cefoxitin, biofilm, *Escherichia coli*, quorum sensing, drug resistance

## Abstract

The study investigates the relationship between biofilm formation and antibiotic resistance in *Escherichia coli* (*E. coli*) isolated from calves. Using biochemical and molecular methods, we identified the isolates and assessed their biofilm-forming ability through an improved crystal violet staining method. The minimum inhibitory concentrations (MICs) of 18 antibiotics against the isolates were determined using the broth microdilution method. The impact of cefoxitin on biofilm formation was analyzed using laser scanning confocal microscopy (LSCM). Additionally, qRT-PCR was employed to evaluate the expression levels of biofilm-related genes (*luxS, motA, fliA, pfs, and csgD*) in response to varying cefoxitin concentrations. Results indicated a significant correlation between antimicrobial resistance (AMR) and biofilm formation ability. Cefoxitin effectively reduced biofilm formation of multidrug-resistant *E. coli* isolates at 1/2 and 1 MIC, with enhanced inhibition at higher concentrations. The QS-related genes *luxS, pfs, motA,* and *fliA* were downregulated, leading to decreased *csgD* expression. At 1/2 MIC, *csgD* expression was significantly reduced. In conclusion, cefoxitin inhibits biofilm formation in multidrug-resistant *E. coli* by down-regulating key genes, offering a potential strategy to mitigate resistance and control infections in calves caused by biofilm-positive *E. coli* isolates.

## STATEMENT OF NOVELTY

The biofilm formation gene and quorum sensing (QS) inhabit the biofilm formation by bacteria, which is significantly related to pathogens’ resistance and virulence. The cefoxitin reduced the adhesion of the drug-resistant strains, which is also a crucial factor in decreasing the *E. coli* pathogenicity.

## Introduction

Calf diarrhea is one of the common diseases in dairy and beef cattle farms. It is reported that the mortality of calves caused by diarrhea in the United States and South Korea is 39% and 53.4% respectively,^1^^,^^2^ whereas in China the mortality rate of calves suffering from diarrhea is between 40% and 50%. *Escherichia coli* (*E. coli*) is one of the main pathogens causing bacterial diarrhea in calves. In recent years, a large number of cases of CD caused by *E. coli* have been reported. Ryu et al. found that 388 strains of *E. coli* were isolated from calf diarrhea samples in Korean, with an isolation rate of up to 56.3%.^2^ And the results of the study by A Springer Browne showed the isolation rate of *Escherichia coli* in calf diarrhea cases among 102 pastures in New Zealand was 20%.^3^ However, in China, the isolate rate of *E. coli* in calf diarrhea cases ranges from 20% to 50%, which varies in different regions.^4^ The relevant research reports indicated that the prevalence of *E. coli* derived from calves is relatively high and plays an important role in the occurrence of calf diarrhea. In Ref.^5^ early research, it was found that cefoxitin has antibacterial activity against 92.3% of Gram negative aerobic bacteria producing β-lactase.^5^ The resistance of *E. coli* strains producing high levels of β-lactase to cephalosporins and results shows that *E. coli* had resistance to 9 different cephalosporins, but was sensitive to cefoxitin.^6^ Cefoxitine has antibacterial activity against *E. coli* that produces β-lactase in both in-vivo and in-vitro. Ref.^7^ mentioned in their study cefoxitin can be considered the preferred cephalosporin drug for infections caused by *E. coli* that produce extended spectrum beta lactases,^7^ and therefore the drug has great potential in the treatment of infectious diseases caused by resistant *E. coli* in calves.

The biofilm formation is related to the development of antibiotic resistance in *E. coli.*^8^^,^^9^ Generally, biofilms enable bacteria to acquire resistance to specific antibiotic agents over extended periods. Therefore, it is necessary to use antibiotics for a long time to treat infections caused by biofilms.^10^ Correlative studies have shown that biofilms have a great role in the spread of antimicrobial resistance. Mobile genetic elements accumulate within biofilms, and movable genetic elements can transfer multidrug resistance genes.^11^ The biofilms create ideal conditions for the horizontal transfer of drug-resistant genes. In bacteria, a biofilm consists of polysaccharides, lipids, proteins, extracellular DNA, carbohydrates, etc., that form an extracellular polymeric substance, which is wrapped around a biological aggregate composed of single or multiple bacterial species to create a highly organized and systematic three-dimensional film-like polymer.^12^ The ability of bacteria to move by flagella increases their ability to interact with surfaces. Bacterial fimbriae, primarily type 1 fimbriae, and curli fimbriae promote surface adhesion once attached.^13^ Recent studies suggested bacterial resistance may be linked to the quorum sensing system (QS).^14^ Bacteria use QS to control different processes related to physiology, including biofilm formation, virulence, swarming, stress survival, motility, and exopolysaccharide synthesis.^15^ It has been proven that the *LuxS*/AI-2 QS system plays a crucial role in *E. coli* biofilm formation and gene expression.^16^ *LuxS*, a member of the activated methyl cycle, affects biofilm development in *E. coli* by stimulating AI-2 metabolism independently.^17^

As a semi-synthetic β-lactam antibiotic derived from cephamycin C, and the antibiotic spectrum of cefoxitin is close to second-generation cephalosporin, and the structure of cephamycins is very similar to cephalosporins. However, the main difference between cefoxitin and other cephalosporins is methoxy groups at the 7 position rather than hydrogen atoms, which confers considerable resistance to hydrolysis by Gram-negative bacteria and *Staphylococcus aureus.*^18^ Like most cephalosporins, cefoxitin binds to penicillin-binding proteins (PBP1A), PBP3, PBP5 and PBP6. The inhibition of PLP resulted in the arrest of peptidoglycan synthesis and bacterial growth. The bactericidal effect of β-lactams is caused by the change of peptidoglycan, which leads to uncontrolled activation of wall autolysin and bacterial lysis.^19^

It is well known that extended-spectrum beta-lactamases-producing *E. coli* are susceptible to cefoxitin.^7^ The previous findings of our research team indicated that cefoxitin had an antibacterial impact on multidrug-resistant *E. coli* from calves that already have biofilms.^18^^,^^20^ In order to explore whether cefoxitin inhibits the formation of the biofilm in *E. coli* derived from calves and its mechanism by which it inhibits biofilm growth, cefoxitin was chosen in the study as a sensitive drug for *E. coli* isolates to clarify how cefoxitin inhibits biofilm formation and genes expression involved in the formation of biofilm in resistant *E. coli* isolates. The findings of this study will provide a theoretical and experimental foundation for future research on resolving the *E. coli* resistance issue and controlling infectious diseases of calves caused by biofilm-positive *E. coli* isolates.

## Materials and methods

### Sample collection

In the current study, 150 Holstein-Friesian calves with diarrhea were selected from a total of 1,000 calves at dairy farms of YiLi Industrial Group Co. Ltd., located near Hohhot in Inner Mongolia, the milk capital of China (latitude: 23.107917818901406, longitude: 113.89809060959752). The samples were usually collected in the morning during 2019 and 2020. Fresh fecal samples from the calves exhibiting diarrhea were inoculated into Eosin-Methylene Blue (EMB) medium and incubated at 37 °C for 24 h.

### Isolates and E. coli culture

The purified single colony was then stained with a gram stain and observed under a microscope to determine the morphology of the bacteria. Biochemical and molecular biological methods identified the suspected isolates. According to the instructions of the biochemical identification tube, an appropriate amount of bacterial solution was inoculated into different biochemical identification tubes. The results of the biochemical reaction were compared with the search table in the biochemical identification coding book of Enterobacteriaceae bacteria, and finally, the names of the bacteria were determined. Then, the DNA of the isolates was extracted as templates according to the instructions of the TaKaRa bacterial genomic DNA extraction kit, and specific gene *uida* (262 bp) of *E. coli* isolates were amplified.^21^ The suspected isolates were confirmed as *E. coli* isolates by biochemical and molecular biological identification.

As a quality control strain for identification and drug sensitivity testing of *E. coli* isolates, *E. coli* ATCC25922 was preserved for experiments. The quality control strain and inoculating isolates into Mueller-Hinton Broth at 37 °C for 5 mL, maintaining the temperature until the logarithmic phase, led to its regeneration. We used the bacterial solution as a seed liquid after adjusting its concentration to 0.5 McFarland turbidity. EMB medium, MH medium and LB broth were obtained from Qingdao Haibo Biotechnology Co., Ltd., while the biochemical identification tubes for *Escherichia coli* were supplied by Hangzhou Tianhe Microbial Reagent Co., Ltd. For molecular biology applications, such as bacterial genomic DNA extraction, PCR kits were purchased from TaKaRa Biotechnology (Dalian) Co., Ltd.

### Biofilm quantification and classification

The ability of biofilm formation of the isolates was determined by staining the crystal violet method. Analytical-grade crystal violet and propidium iodide (purity ≥98%) were purchased from Solarbio Biotechnology Co., Ltd. When the isolates had been regenerated, colonies were taken and inoculated into 5 mL lysogeny broth (LB), with the solution adjusted to 0.5 McFarland. A 10% inoculum concentration was used to inoculate 96 wells with the isolate. Incubation was followed by washing with PBS and fixing with 99% methanol for 15 min, then drying. We stained the plates with 2.5% crystal violet for five minutes and washed them to remove the unbound dye. Microplate readers were used to measure the OD570 value in triplicate. Averaging the readings per well was then performed. The biofilm forming ability: critical value (ODC570) = mean value of blank control + (3 × standard deviations of blank control). When OD570 ≤ ODC570, it was judged that bacteria could not form biofilm; when ODC570 < OD570 ≤ 2ODC570, it was judged that bacteria formation of ability was weak; when 2ODC570 < OD570 ≤ 4ODC570, it was judged that bacteria biofilm forming ability was medium; when 4ODC570 < OD570, it was judged that bacteria biofilm forming ability was strong. In the experiments, XTT bacterial proliferation and toxicity detection reagent KGA313-1 from Jiangsu Kaiji Biotechnology Co., Ltd were utilized.

### Antibacterial susceptibility determination

The broth microdilution method determined the minimum inhibitory concentrations (MICs) of 18 antibiotics against *E. coli* isolates. The following antimicrobial agents (18) were used in this study, Tetracyclines (Doxycycline, Tetracycline, Minocycline); Quinolones (Ciprofloxacin, Levofloxacin, Gatifloxacin, Ofloxacin); Aminoglycosides (Amikacin, Gentamicin, Kanamycin, Tobramycin); Cephalosporins (Cefalotin, Cefoxitin, Cefotaxime, Cefepime); Penicillins (Ampicillin); Folic acid antagonists (Trimethoprim, Sulfadiazine). A total of 18 antibacterial standard drugs were bought from the China Veterinary Drug Administration. A fresh dilution of all compounds was prepared daily, dissolved, and diluted. Microdilution was used to determine the antibacterial minimum inhibitory concentrations (MICs) against *E. coli* isolates on the Mueller–Hinton medium. The bacterial solution was diluted with approximately 0.5 McFarland and again diluted to 1 × 10^5^ CFU/mL by turbidimetry with a spectrophotometer. According to the determined MIC value of the antibiotics agent on the isolates and ATCC 25922 strain, the antibiotics agent was diluted to different concentrations with MH broth. A quality control test was conducted using reference *E. coli* ATCC25922 for antibacterial susceptibility testing. In the current research, an in-vitro drug susceptibility test of the *E. coli* isolates was performed according to the antimicrobial susceptibility test executive standard (CLSI 2021 protocol^22^).

### Biofilm adhesion value (B) determination

The LB or MH broth was used for adjustment, and the bacterial solution was diluted with approximately 0.5 McFarland and again diluted to 1 × 10^5^ CFU/mL. According to the determined MIC value of the antibiotics agent on isolates and ATCC 25922 strain, the antibiotics agent was diluted to different concentrations with MH broth. The plates were incubated for 24 h at 37 °C followed by determining the OD595 value (A1). We discarded the excess liquid, washed the plates with PBS, and dried the plates. 1% crystal violet dye was used to stain the plates for 30 min, after washing and drying, 95% (v/v) ethanol was used to dissolve the dye. In addition, microplate readers were used to measure the OD595 value (A2). The adhesion value B is calculated as follows: B = (A2-A2C)/(A1-A1C) (C: the value of the negative control group).

### Cefoxitin impact on the formation of biofilm by E. coli

High-resistance isolates were chosen and seeded in 96-well plates at 2MIC, MIC, 1/2 MIC, and 1/4 MIC concentrations to detect biofilm formation. LB was also used as a negative control. An inoculum concentration of 100 microliters was applied to each well after adjusting the bacterial concentration to 0.5 McFarland. The plates were incubated for 0, 2, 4, 6, 8, 10, 12, 16, 24, 36, 48, and 60 h at 37 °C, and after 24 h the medium was replaced. The 100 µL of LB broth medium and 20 µL of XTT (Keygentec, China) were inoculated into the per well. The plates were incubated for 2 h at 37 °C, and the OD450 value was determined after the incubation. Ae = A − An (Ae: the OD450 value of the experimental group; A: the OD450 value of each test hole; An: the OD450 value of the negative control group)

### Laser scanning confocal microscopy observation

The effect of cefoxitin on the formation of biofilms was evaluated by LSCM. It was decided to use the strain (strain 38) that formed the most biofilms in this experiment. On a 24-well cell culture plate, pieces of cell slides treated with UV were placed overnight, and a culture solution containing different concentrations of the antibiotics agent was added. LB was used in the blank group, where 1 mL inoculum was added to each well after adjusting the bacterial concentration to 0.5 McFarland. We used a constant temperature incubator to incubate the 24-well plate for 24 and 48 h at 37 °C. The medium was discarded after incubation. Afterward, PBS was used to wash the cell slides and fixed them with glutaraldehyde for 1.5 h. Later, PBS was again used, and the slides were rinsed to remove the fixative and stained with Fluorescein Isothiocyanate (Maokangbio, China) in the darkroom for 30 min at 25 °C. The cell slides were washed with PBS after staining. After staining the cell slides in the dark room for 15 min at 37 °C, these were stained with Propidium Iodide (Solarbio, China). A Laser Scanning Confocal Microscope 800 (Zeiss, Germany) was used to observe the slides after washing them in PBS and sealing them with autofluorescence quenching.

After treatment with cefoxitin, the coverslips formed biofilms with the drug-resistant strain 38. The polysaccharide matrix of the bacteria biofilms is labeled by FITC-ConA, which emits green fluorescent light at 519 nm, while PI labels the nucleus of the bacteria cells and discharges red fluorescence at 617 nm excitation wavelength. An orange-yellow hue is produced when the fluorescences are superimposed. Based on LSM 800 results, the blank control prepared a highly adhering biofilm and fluoresced green.

### Biofilm gene expression

The effects of different concentrations of cefoxitin on the expression levels of biofilm-related genes *luxS, motA, fliA, pfs,* and *csgD* were detected by qRT-PCR. First, the antibiotic agent was diluted to different concentrations with MH broth. The drug-resistant strain 38 was incubated for 24h and 48h with cefoxitin of various concentrations. After treatment with cefoxitin of different concentrations, the total RNA of the strain was extracted from the *E. coli* culture using the TRIzol (Takara) method. The reverse transcription kit contained instructions for generating a cDNA template, and gDNA was eliminated from total RNA before synthesis of the cDNA template. Quantitative Reverse Transcription-PCR (qRT-PCR) was used as per manufacturer guidelines. The internal reference gene *gap*A and six primers^23^^,^^24^) from BGI Technology were used (Table 1). RNA extraction, reverse transcription, and qPCR, reagents were purchased from TaKaRa Biotechnology (Dalian) Co., Ltd.

**Table 1.** Details of qRT-PCR primer sequences.

Gene	Primers (5′–3′)	Length (bp)	References	Accession No.
*luxS*-F	TGCCACACTGGTAGACGTTC	116	^24^	
*luxS*-R	TGATTGGTACGCCAGATGAG			P45578
*fliA*-F	GCTGGCTGTTATTGGTGTCG	112	^24^	
*fliA*-R	CAACTGGAGCAGGAACTTGG			EG11355
*csgD* **-F**	CCGCTTGTGTCCGGTTTT	97	^23^	
*csgD*-R	GAGATCGCTCGTTCGTTGTTC			ECK1026
*motA*-F	CTTCCTCGGTTGTCGTCTGT	120	^24^	
*motA*-R	CTATCGCCGTTGAGTTTGGT			P09348
*pfs*-F	CGGCAACAGCCAGGAACTCA	169	^24^	
*pfs*-R	GCGAAAATCCGCCACAACTT			NP_414701.1
*gapA*-F	GAAATGGGACGAAGTTGGTG	104	^24^	
*gapA*-R	AACCACTTTCTTCGCACCAG			P0A9B2

### Data analysis

The targeted genes relative expressions were measured through 2-ΔΔCt (where ΔCt = Ct (target gene) − Ct(reference gene), ΔΔCt = ΔCt (test) − ΔCt (calibrator) as mentioned earlier. SPSS 19.0 (SPSS Chicago, IL) analytical tool was used for statistical analysis. Different symbols, such as * and **, were used for significant differences at *p* ≤ .05 and *p* ≤ .01, respectively.

## Results

### Biofilm classification

In biofilm classification, 31.4% (16/51) of isolates have no ability to form biofilms, while a total of 68.6% (35/51) of isolates are capable of forming biofilms. The *E. coli* biofilm-positive isolates were divided into three groups with strong, moderate, and weak capabilities for the formation of biofilms 37.1% (13/35), 25.7% (9/35), and 37.1% (13/35), respectively.

### Antibacterial susceptibility determination of E. coli biofilm isolates

The results of the antibacterial susceptibility are mentioned in Table 2. The eleven isolates of *E. coli* were multidrug-resistant. Among them five isolates were resistant to 12–16 antibiotics (six categories), three isolates resistant to 8–11 antibiotics (five categories), two isolates resisant 5–8 antibiotics (four categories) and one isolate resistant to 4 antibiotics (three categories). However, only two isolates were not multidiug-resistant and resistant to 3 antibiotics (two categories).

**Table 2.** Antimicrobial resistance phenotype of isolates with strong biofilm-formation ability.

Isolates	Antimicrobial resistance phenotype (number) (categories)
#38	Doxycycline, Tetracycline, Minocycline, Ciprofloxacin, Levofloxacin, Gatifloxacin, Ofloxacin, Gentamicin, Kanamycin, Tobramycin, Ampicillin, Cefalotin, Cefepime, Cefotaxime, Sulfadiazine, Trimethoprim (16) (6)
#58	Doxycycline, Tetracycline, Minocycline, Ciprofloxacin, Levofloxacin, Ofloxacin, Gentamicin, Kanamycin, Ampicillin, Cefoxitin, Cefalotin, Cefotaxime, Sulfadiazine, Trimethoprim (14) b(6)
#64	Doxycycline, Tetracycline, Ciprofloxacin, Levofloxacin, Gatifloxacin, Ofloxacin, Gentamicin, Kanamycin, Ampicillin, Cefalotin, Cefepime, Cefotaxime, Sulfadiazine (13) (6)
#3	Doxycycline, Tetracycline, Minocycline, Ciprofloxacin, Levofloxacin, Gatifloxacin, Kanamycin, Ampicillin, Cefalotin, Cefepime, Cefotaxime, Sulfadiazine, Trimethoprim (13) (6)
#10	Doxycycline, Tetracycline, Ciprofloxacin, Levofloxacin, Gentamicin, Kanamycin, Ampicillin, Cefalotin, Cefepime, Cefotaxime, Sulfadiazine, Trimethoprim (12) (6)
#26	Doxycycline, Tetracycline, Ciprofloxacin, Levofloxacin, Gatifloxacin, Ampicillin, Cefalotin, Cefepime, Cefotaxime, Sulfadiazine, Trimethoprim (11) (5)
#45	Doxycycline, Tetracycline, Minocycline, Ciprofloxacin, Levofloxacin, Gatifloxacin, Ofloxacin, Gentamicin, Ampicillin, Sulfadiazine, Trimethoprim (11) (5)
#12	Tetracycline, Ciprofloxacin, Levofloxacin, Gatifloxacin, Gentamicin, Ampicillin, Sulfadiazine, Trimethoprim (8) (5)
#2	Doxycycline, Tetracycline, Ampicillin, Cefalotin, Cefepime, Cefotaxime, Sulfadiazine, Trimethoprim (8) (4)
#43	Tetracycline, Ciprofloxacin, Levofloxacin, Trimethoprim, Cefepime (5) (4)
#8	Doxycycline, Tetracycline, Ampicillin, Trimethoprim (4) (3)
#14	Tetracycline, Sulfadiazine, Trimethoprim (3) (2)
#15	Tetracycline, Sulfadiazine, Trimethoprim (3) (2)

### Biofilm adhesion value (B)

As shown in Table 3, except for isolate#10, the 1 MIC, 1/2 MIC, and 1/4 MIC levels of cefoxitin showed highly significant inhibition of the adhesion value of 6 isolates. The 1/8 MIC level of cefoxitin displayed highly significant inhibition of the adhesion values of isolates #38, #58, and #45 and significantly inhibited the adhesion values of 2 isolates. It appears that cefoxitin is the most apparent inhibitor of the adhesion value of isolates 38 because it had the most significant effect on the drug-resistant isolate 38.

**Table 3.** Adhesion value of 7 antimicrobial-resistance biofilm isolates treated with different concentrations of cefoxitin (′X ± S).

Isolate Concentration	1 MIC	1/2 MIC	1/4 MIC	1/8 MIC	0
#38	2.600 ± 0.497**	2.550 ± 0.339**	2.073 ± 0.333**	4.240 ± 0.794**	8.842 ± 0.621
#3	3.102 ± 0.526**	1.735 ± 0.264**	1.479 ± 0.240**	5.736 ± 0.148*	7.043 ± 0.575
#58	1.833 ± 0.118**	2.049 ± 0.193**	2.376 ± 0.089**	3.210 ± 0.085**	4.898 ± 0.577
#64	2.676 ± 0.364**	1.792 ± 0.181**	2.691 ± 0.067**	3.736 ± 0.286	4.320 ± 0.612
#10	4.235 ± 0.284	2.887 ± 0.997**	2.088 ± 0.493**	4.585 ± 0.814	5.816 ± 0.072
#45	3.464 ± 0.249**	0.963 ± 0.072**	1.390 ± 0.140**	2.805 ± 0.081**	4.708 ± 0.472
#26	1.185 ± 0.636**	1.193 ± 0.452**	1.807 ± 0.558**	2.537 ± 0.559*	4.193 ± 0.023

Note: (′X ± S)

** *p ≤* .01, & 0 MIC Control; (′X ± S)

* 0.05 *≤ p ≤ .01*, & 0 MIC Control.

### Effect of cefoxitin on biofilm formation by E. coli

**Figure 1.**
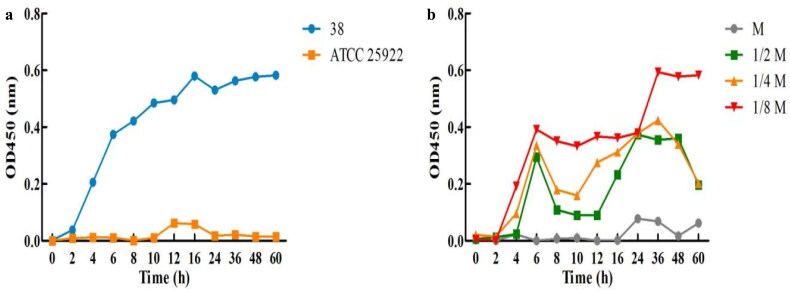
Biofilm growth curve of *E. coli* treated with cefoxitin. (a) Untreated group and (b) Treated group.

According to Fig. 1. A, the biofilm growth curve of stain 38 *E. coli* in the blank group exposed to the lag period reaches a logarithmic phase after 2–6 h, a peak phase after 16 h, and a stationary phase after 36 h. As shown in Fig. 1. B, cefoxitin displayed a significant repressive effect on the biofilm generation of *E. coli*. It can be seen that cefoxitin has the most potent inhibitory effect on biofilm growth at MIC and the weakest inhibitory effect at 1/8 MIC. Cefoxitin exhibited dose-dependent inhibitory effects on biofilm growth.

### Laser scanning confocal microscopy observation

**Figure 2.**
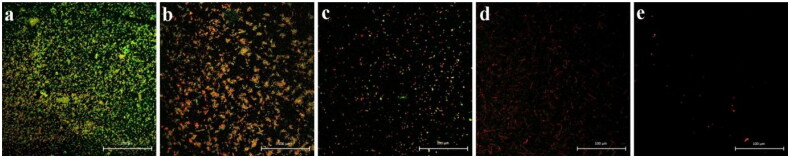
Comparison of inhibitory effects of cefoxitin on biofilm formation of *E. coli* in 24 h culture. (a) Untreated group, (b) 1/8 MIC concentration-treated group, (b) 1/4 MIC concentration-treated group, (d) 1/2 MIC concentration-treated group, and (e) MIC concentration-treated group.

**Figure 3.**
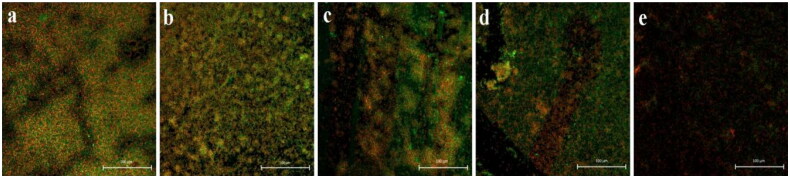
Comparison of inhibitory effects of cefoxitin on biofilm formation of *E. coli* in 48 h culture. (a) Untreated group, (b) 1/8 MIC concentration-treated group, (c) 1/4 MIC concentration-treated group, (d) 1/2 MIC concentration-treated group, and (e) MIC concentration-treated group.

As shown in Figs. 2 and 3, cefoxitin’s MIC concentration caused the most significant reduction in biofilm formation in *E. coli*, followed by 1/2 MIC, 1/4 MIC, and 1/8 MIC. Figure 3 shows that the inhibition effects of cefoxitin on biofilm formation of *E. coli* in 24 h culture by 1/2 MIC and MIC level was most patent. The strain treated with MIC concentration completely suppressed the biofilm formation and significantly decreased the cell number. However, as shown in Fig. 3, the suppression of cefoxitin on the formation of biofilm of *E. coli* in 48 h culture was not noticeable.

### Effect of cefoxitin on the expression of genes related to biofilm generation

**Figure 4.**
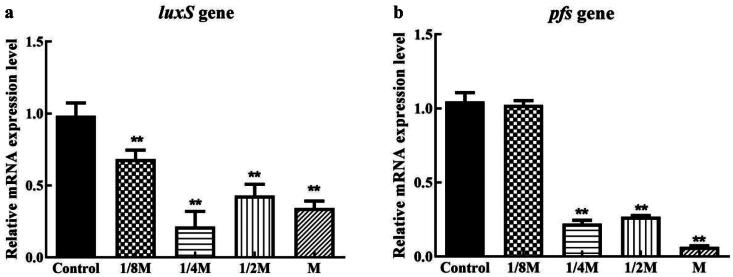
Effects of cefoxitin on the relative expression of the *luxS* and *pfs* genes in AI-2 synthesis (** indicates a significant difference at *p* ≤ .01).

**Figure 5.**
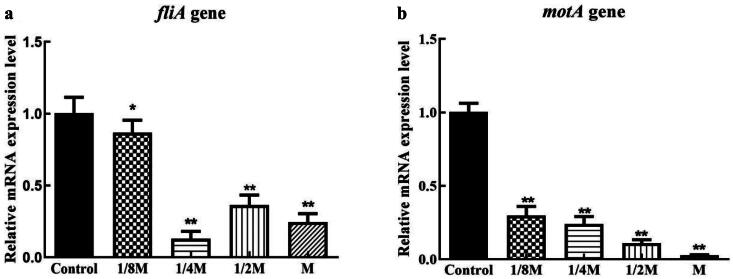
Effects of cefoxitin on the relative expression of the *fliA* and *pfsmotA* genes (** indicates a significant difference at *p ≤* .01).

**Figure 6.**
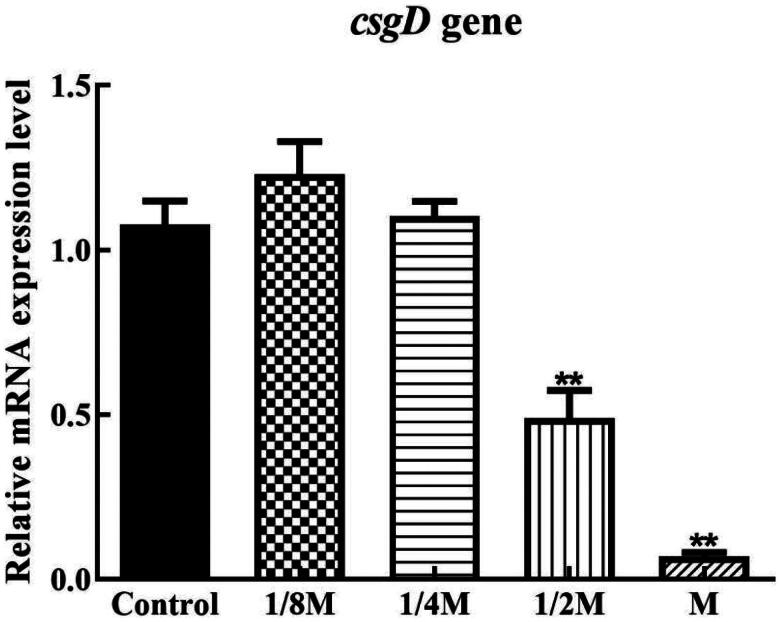
Effects of cefoxitin on the relative expression of the *csgD* gene (** indicates a significant difference at *p ≤* .01).

As shown in Fig. 4, the down-regulation of the *luxS* gene by cefoxitin treatment was prominent, and it was observed that all treated groups differed markedly from the untreated group. At 1/4 MIC, 1/2 MIC, and MIC levels, the relative expression of the *pfs* gene was significantly down-regulated and significantly different from the blank group. Figure 5 shows that all the treated groups showed down-regulated expression levels of the *fliA* gene, but the inhibitory effect of cefoxitin at the 1/4 MIC, 1/2 MIC, and MIC concentrations with MIC concentration showed the most significant impact. Also, the down-regulation of the *motA* gene by cefoxitin treatment was obvious, and all treated groups showed significant differences from untreated groups, and *motA* gene expression was barely detected after cefoxitin treatment at the MIC level. Figure 6 shows that only the 1/2 MIC and MIC concentrations showed down-regulation of the *csgD* gene expression.

## Discussion

The negative impact of AMR on humans, increasing daily, was recognized by the World Health Organization and major national organizations over the past decade. It was estimated in a 2016 report that if the current evolution and spread of AMR don’t stop, it will cause 10 million excess deaths worldwide and cost US$100 trillion by 2050.^25^ At the same time, drug-resistant pathogens can be transmitted through animal food/environment-human bodies. The increasing number of resistant strains of *E. coli* has seriously threatened human food hygiene and public health safety.^26^ The current research evaluated whether antibacterial agents would inhibit *E. coli* biofilm formation to reduce *E. coli* survivability and subsequent pathogenicity.

Our results found that 92.2% of *E. coli* isolates were multi-drug resistant (MDR), and this is following the previous studies in China.^27^ The *E. coli* isolates showed high resistance to ampicillin, tetracycline, and trimethoprim, agreeing with the results of the other relevant research in China and other countries.^27^ Our results found that the gentamicin resistance rate of the isolates was 51%, which was different from the gentamicin resistance rate (3.7%) reported by Srivani et al. In the treatment of colibacillosis of calves in different regions, the choice of antibiotics is different, resulting in different resistance of *E. coli* to antibiotics.^28^ The results in this study showed that *E. coli* isolates had the same resistance rate to gentamicin and kanamycin (51.0%) and the same resistance rate to cefotaxime and cefotaxime (68.6%), which indicated that there was cross-resistance between *E. coli* isolates.

The results showed that 94.3% (33/35) of the biofilm-positive isolates were multi-drug resistant, 18 (51.4%) isolates of the biofilm-positive strains were resistant to 6 categories of antibiotics, and the drug resistance spectrum of the biofilm-positive isolates was broader than non-biofilm isolates (Fig. 1), which was consistent with Ito, A. et al.’s report that biofilm isolates of *E. coli* are more resistant than non-biofilm isolates.^29^ Through experimental studies, Scudamore et al. and Anderl et al. also confirmed that antibiotics could not penetrate the biofilm or only a small amount of antibiotics could penetrate the biofilm and act on the bacteria, leading to reduced or even inactivation of the activity of antibiotics against the membranous bacteria.^30^^,^^31^

Our research displayed that the drug resistance rate of biofilm isolates to ciprofloxacin and levofloxacin was greater than that of non-biofilm isolates (*p* ≤ .05) (Table 4), which was consistent with the report by Butt and Khan^32^ that biofilm-positive isolates easily became resistant to fluoroquinolone antibiotics. In *E. coli*, reversible adhesion is the initial stage of biofilm formation. When membrane-bound sensory proteins are stimulated to be produced, cells attached reversibly to surfaces now produce extracellular polymeric substances that allow the cells to form a cell-to-cell bridge that binds them irreversibly to the surface. Thus, adhesion is the basis of biofilm formation. Current research findings displayed that cefoxitin could reduce the adhesion value of biofilm-positive isolates (Table 3).

**Table 4.** The resistance of biofilm positive and negative *E. coli* isolates to the 18 antimicrobial agents.

Antibiotics	resistance rate of all isolates % (*n* = 51)	resistance rate of biofilm positive isolates % (*n* = 35)	resistance rate of biofilm negative isolates % (*n* = 16)	Chi-square value	*p*
Doxycycline	70.6 (36/51)	68.6 (24/35)	75 (12/16)	0.219	.892
Tetracycline	98.0 (50/51)	100 (35/35)	87.5 (14/16)	4.554	.094
Minocycline	19.6 (10/51)	22.9 (8/35)	12.5 (2/16)	0.747	.628
Ciprofloxacin	53.0 (27/51)	62.9 (22/35)	31.3 (5/16)	4.403	.036
Levofloxacin	58.8 (30/51)	68.6 (24/35)	37.5 (6/16)	4.377	.036
Gatifloxacin	45.1 (23/51)	51.4 (18/35)	31.3 (5/16)	1.806	.179
Ofloxacin	33.3 (17/51)	40 (14/35)	12.5 (2/16)	3.857	.050
Amikacin	11.8 (6/51)	8.6 (3/35)	18.8 (3/16)	1.096	.563
Gentamicin	51.0 (26/51)	51.4 (18/35)	50 (8/16)	0.009	.925
Kanamycin	51.0 (26/51)	51.4 (18/35)	50 (8/16)	0.009	.925
Tobramycin	21.6 (11/51)	20 (7/35)	25 (4/16)	0.162	.971
Cefalotin	68.6 (35/51)	65.7 (23/35)	68.8 (11/16)	0.046	.831
Cefoxitin	9.8 (5/51)	11.4 (4/35)	6.3 (1/16)	0.333	.944
Cefotaxime	68.6 (35/51)	68.6 (24/35)	68.8 (11/16)	0.000	.990
Cefepime	51.0 (26/51)	51.4 (18/35)	50 (8/16)	0.009	.925
Ampicillin	84.3 (43/51)	88.6 (31/35)	75 (12/16)	1.529	.411
Trimethoprim	90.2 (46/51)	88.6 (31/35)	87.5 (14/16)	0.012	1.000
Sulfadiazine	78.4 (40/51)	82.9 (29/35)	68.8 (11/16)	1.292	.441

The results of the growth curve showed that cefoxitin could effectively inhibit the growth rate of biofilm, and the inhibition effect was proportional to the concentration of cefoxitin (Fig. 1). The results of the LSCM examination showed that cefoxitin could effectively inhibit the biofilm formation of *E. coli* after 24 h culture, and the inhibition effect was also proportional to the concentration of cefoxitin (Fig. 2). This was consistent with a report that cefoxitin could significantly inhibit the formation of staphylococcus biofilm in a dose-dependent manner.^33^ In conclusion, cefoxitin can effectively reduce the production of biofilm. The reduction of biofilm production can decrease bacterial resistance caused by biofilm formation, inhibit persistent infection caused by biofilm, and reduce the harm brought by biofilm-positive isolates of *E. coli* to animals and humans.^34^

**Figure 7.**
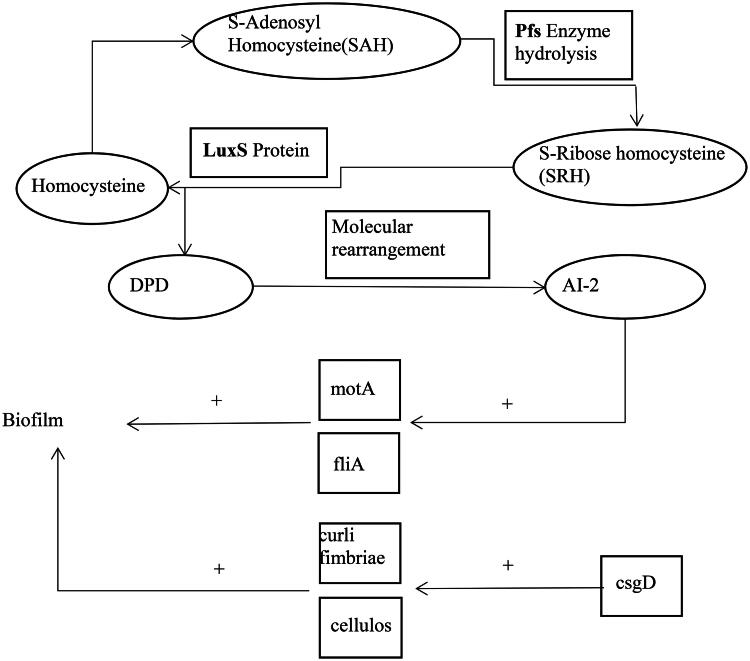
Model for the five gene regulation of biofilms.

The study results showed that cefoxitin could significantly down-regulate the expression of the *csgD* gene in 1/2 MIC and MIC. *CsgD* controls two determinants of bacterial adhesion and biofilm formation, signaling bacteria secrete substances called auto-inducers (AI) to communicate with each other by binding to receptor proteins and causing bioluminescence.^35^ It is reported that the *LuxS*/AI-2 system should stimulate biofilm formation.^14^ Autoinducer 2, as a post-transcriptional factor, regulates bacterial biofilm formation.^36^ As shown in Fig. 7, as part of the *LuxS*/AI-2 synthesis, the enzyme SAH hydrolase (SahH) converts S-adenosylhomocysteine (SAH) to homocysteine either in a one-step reaction or in a two-step reaction using *Pfs* and *LuxS*. By cleaving the thioether linkage of SRH, *LuxS* produces 4,5 dihydroxy-2,3-pentanedione (DPD), which is capable of rearranging into R- or S-2-methyl-2,3, 3,4-tetrahydroxytetrahydrofuran (R-or S-THMF).^14^ When the concentration of AI-2 in the extracellular matrix of the biofilm reaches its limit, it will trigger a series of signal transduction, leading to changes in the behavior of the bacterial population.^24^ The *fliA* controls the operon *pfsmotA*, which also affects biofilms’ formation. Our study shows that cefoxitin significantly lowered *pfs* gene expression at 1/4 MIC, 1/2 MIC, and MIC and *luxS* gene expression at 1/8 MIC, 1/4 MIC, 1/2 MIC, and MIC. The cefoxitin at the 1/4 MIC, 1/2 MIC and MIC significantly down-regulated the expression of genes related to the QS system.^24^ In this study, the expression of the flagella gene (*fliA*) and flagellar motion gene (*pfsmotA*) regulated by QS was detected. A significant reduction in the expression of the *fliA* gene was observed at the 1/4, 1/2, and MIC concentrations of cefoxitin, while the production of *pfsmotA* was significantly reduced at 1/8, 1/4, 1/2, and MIC concentrations of cefoxitin. The results revealed that cefoxitin at the 1/4 MIC, 1/2 MIC, and MIC not only significantly down-regulated the expression of QS system-related genes, but also significantly down-regulated *fliA* and *pfsmotA* genes, which were regulated by QS. Cefoxitin’s ability to inhibit QS initiation at lower concentrations is significant. AMR can be prevented by inhibiting bacterial QS, a new and promising antimicrobial strategy.

Among the target genes regulated by *csgD*, the *csgD* gene activates transcription of the csgBAC operon encoding a structural subunit of frizzi and transcription of the *adrA* gene, a positive effector of cellulose biosynthesis. The csgBAC operon is used to synthesize, secrete, and assemble frizzy pili components to promote the production of frizzi. At the same time, the *adrA* gene encodes the *AdrA* proteins that catalyze the synthesis of cycloid-GMP, which stimulates cellulose production.^37^ The *csgD* gene has a positive regulatory effect on the formation of biofilms. The results showed that cefoxitin can significantly down-regulate the expression of the *csgD* gene at the 1/2 MIC and MIC. By down-regulating the expression of the *csgD* gene, cefoxitin reduced the synthesis of curly pili and cellulose of *E. coli* isolates from calves and inhibited the adhesion of the isolate. Thus, it has a negative effect on the formation of biofilms. The study’s results proved that cefoxitin inhibits biofilm formation by downregulating the expression of the *csgD* gene.

As a semi-synthetic β- lactam antibiotic, the antibacterial mechanism of cefoxitin is the same as that of all β- lactam drugs. Cefoxitin exerts an antibacterial effect by inhibiting peptidoglycan synthesis in the bacterial wall. The bacteria are easy to have drug resistance against for the sake of their single characteristic of the antibacterial mechanism of cefoxitin, are easily produced bacteria drug resistance. The Chinese medicinal herbs have the characteristics of being less poisonous and having side effects, lower drug residue, and difficulty in making bacteria tolerant to the drug. In the future, cefoxitin can be used with Chinese medicinal herbs or monomers to control and treat infections caused by biofilm-positive *E. coli* isolates in clinics. They would have complementary advantages and achieve synergistic inhibition of biofilm-forming bacterial isolates, which provides a new direction for our future research.

QS system controls bacterial biofilm formation, which is highly related to the virulence and resistance of pathogens. In the present study, as shown in Fig. 7, the effect of cefoxitin on biofilm formation and QS-related gene expression of AMR *E. coli* strains isolated from Holstein-Friesian calves with diarrhea was investigated by LSCM observation and qRT-PCR. As a semi-synthetic β- lactam antibiotic, the antibacterial mechanism of cefoxitin is the same as that of all β- lactam drugs. Cefoxitin exerts an antibacterial effect by inhibiting peptidoglycan synthesis in the bacterial wall. The bacteria are easy to have drug resistance against for the sake of their single characteristic of the antibacterial mechanism of cefoxitin, are easily produced bacteria drug resistance. The Chinese medicinal herbs have the characteristics of being less poisonous and having side effects, lower drug residue, and difficulty in making bacteria tolerant to the drug. Therefore, more studies are needed to observe the combined use of cefoxitin and traditional Chinese medicine monomers on the biofilm formation and growth of *E. coli Escherichia coli* from calves with diarrhea. In addition, we need to know if cefoxitin can be used with Chinese medicinal herbs or monomers to control and treat infections caused by biofilm-positive *E. coli* isolates in clinics and if they have complementary advantages and achieve synergistic inhibition of biofilm-forming bacterial isolates, which would provides a new direction for our future research.

## Conclusion

The study concluded that biofilm-forming *E. coli* isolates are more resistant to drugs than non-biofilm isolates. Cefoxitin effectively reduce biofilm adhesion and inhibit biofilm formation in *E. coli,* with its effectiveness increasing at higher concentrations. It works by down-regulating key genes involved in biofilm formation, such as *luxS, pfsmotA, fliA, pfs,* and *csgD*. These findings provide a deeper understanding of the link between biofilm formation and drug resistance in *E. coli* from diarrheic calves and offer a foundation for future research aimed at tackling *E. coli* resistance and controlling related infectious diseases in calves.

## Data Availability

A reasonable request can be made to the corresponding author for all materials, data, and protocols that support the findings of this study.
